# Characterizing the bacterial surface profiles of menstrual cups and their association with user characteristics and vaginal microbiomes in an adolescent cohort from western Kenya

**DOI:** 10.12688/gatesopenres.16380.1

**Published:** 2026-05-08

**Authors:** Garazi Zulaika, Rachel Nordgren, Walter Agingu, Adit Chaudhary, Ezekiel Dibondo, Patriciah Wambua, Anna Maria van Eijk, Laura Rusie, Ankur Naqib, Fredrick Otieno, Penelope A. Phillips-Howard, Supriya D. Mehta

**Affiliations:** 1 Liverpool School of Tropical Medicine Department of Clinical Sciences, Liverpool, England, UK; 2 University of Chicago, Chicago, Illinois, USA; 3 Nyanza Reproductive Health Society, Kisumu, Kenya; 4 Rush University Medical Center, Chicago, Illinois, USA; 5 Rush University Medical Center, Chicago, Illinois, USA; 6 Rush University College of Medicine, Chicago, Illinois, USA

**Keywords:** Menstrual cups, microbiome, vaginal health, bacterial vaginosis, putative pathogens, adolescent girls, low-resource settings, WASH

## Abstract

**Background:**

Menstrual cups are increasingly promoted in low-resource settings as long-lasting and cost-effective menstrual hygiene solutions. However, no studies have been done among cup users to characterize the bacterial communities found on cups with long-term use when stored. This study sought to comprehensively characterize the bacterial surface profile of the menstrual cup, identify factors associated with putative pathogens, and quantify the vaginal microbiome as potential source for menstrual cup bacterial communities.

**Methods:**

Over 30 months of follow-up, 369 menstrual cup samples were collected from 172 secondary schoolgirls participating in a randomized controlled trial in western Kenya. Samples were obtained from cups brought to school by girls during study visits. Menstrual cup and vaginal microbiomes were assessed using 16S rRNA gene amplicon sequencing. Mixed effects models were applied to identify factors associated with putative pathogens ( *Escherichia coli*, *Staphylococcus aureus*, coliform bacteria), and factors associated with vaginal microbiome as potential source environment to bacterial surface profile of the menstrual cup, estimated via fast expectation-maximization for microbial source tracking (FEAST).

**Results:**

Menstrual cup bacterial surface profile composition was primarily comprised of soil and water bacteria. However, taxa specific to the vaginal microbiome (e.g., *Lactobacillus crispatus*, *L. iners*, and *Gardnerella vaginalis*) were also recovered from cups. The mean relative abundance (presence) of *E. coli* and *S. aureus* was 0.09% (36%) and 0.36% (24%), respectively, with higher relative abundance among participants with HSV-2 or non-optimal vaginal community state type 4. Damaged cups were also associated with higher relative abundance of putative pathogens, while antibiotic use was inversely associated with *E. coli.* On average, 25.1% of cup microbiota originated from the vagina, with lower contributions among older participants and those with damaged cups. No serious adverse events related to the menstrual cup were observed in the cohort.

**Conclusions:**

Interventions focused on improved cleaning and storage tools and methods, and access to clean water and sanitation infrastructure are required to maximize safety of menstrual cup use in low-resource settings.

## Background

The menstrual cup is a menstrual hygiene management product that is inserted vaginally to collect menstrual blood. While it remains less well-known than other menstrual hygiene products (e.g., sanitary pads, tampons), it has a long history, a strong safety profile, and can be used for 10 years before needing replacement, making the cup a long-lasting and cost-effective menstrual health investment. ^1^^,^^2^

Over the past decade, menstrual cups have gained traction in low-resource settings as a sustainable product solution for menstrual health and hygiene (MHH). ^3^ Recent research has found that, beyond being a practical and acceptable menstrual management option, ^4^^–^^9^ menstrual cups may also serve as a multipurpose tool to reduce the risk of bacterial vaginosis (BV) and sexually transmitted infections (STI). ^10^^–^^12^ Studies indicate that menstrual cups have no adverse effect on the vaginal microflora, help maintain a *Lactobacillus-*dominated vaginal microbiome (VMB), and reduce rates of BV. ^10^^,^^11^^,^^13^ In a cluster-randomized study of 436 secondary schoolgirls in western Kenya, cup recipients had 37% increased odds of optimal *L. crispatus* dominated Community State Type 1 (CST-1) (95% CI: 1.06 – 1.75) and a 24% lower odds of BV (95% CI: 0.59 – 0.98). ^10^ The study also found a higher rate of STI among usual practice controls at 30-months follow-up compared to menstrual cup arm participants (18.1% vs. 13.5%). ^10^ Another study among primary school students found a 35% reduction in BV prevalence among those given a menstrual cup (13%) compared to pad recipients (20%) and controls (19%). ^11^ A randomized controlled trial in 4,137 secondary schoolgirls found a 33% (95% CI: 5% – 53%) lower incidence of HSV-2 among girls who received menstrual cups relative to a control group. ^12^ These data suggest menstrual cups could be a low-cost, multipurpose MHH tool for women at high risk of sexual and reproductive health harms, including STI and HIV.

While menstrual cups are increasingly promoted in low-resource settings for their reusability, long functional life, and low environmental impact, ^14^ their safety profile remains uncertain for populations living in these settings. Limited access to clean water for cleaning and inadequate hygiene and sanitation facilities during menstruation have been shown to increase risk of urinary tract infections and BV. ^15^^–^^17^ In these settings, it is possible that inadequate cup hygiene could introduce external bacteria and elevate infection risk. However, to date little is known about the bacterial surface profile of the menstrual cup and its relation to cup users’ vaginal health. In a single study in Kenyan adolescent girls, *Escherichia coli* growth was detected on 37.1% of participants’ cups (95% CI: 21.1% – 53.1%) via bacterial cultivation, with higher detection among new cup users than among established users (53% vs. 22.2%, p = 0.12), suggesting contamination was more frequently due to handling than persistent colonization. ^18^ A small number of studies have used *in vitro* bacterial cultivation methods to capture bacterial abundance on menstrual cups. One such study by Wunsch and colleagues (2022) assessed the effectiveness of common cleaning procedures by capturing the elimination of bacteria, specifically *Staphylococcus aureus,* from silicone menstrual cups. ^19^ A second *in vitro* study by Schlievert and colleagues (2019) found no enhanced *S. aureus* growth or TSST-1 production on non-absorbent devices, including menstrual cups, while they did on absorbents like tampons. ^20^

While these *in vitro* studies provide important mechanistic insights into how materials interact with specific bacterial species under controlled conditions, *in vivo* user studies are needed to evaluate conditions that are physiologically relevant to the human vaginal environment and to capture the effect of human behavior in different settings. These are especially important for items like menstrual cups, where local menstrual practices and taboos, and cumulative handling, storage, and environmental exposures over time may shape microbial profiles in ways not observable in laboratory models. For example, real-world cleaning practices may significantly differ by context and dramatically affect the cups’ condition. ^1^^,^^21^ Additionally, menstrual blood contains enzymes and nutrients that can influence bacterial growth which may be hard to substitute in the lab, and biofilm formation on cups *in vitro* may not translate to biofilm formation in real life or to real infection risk in users. ^22^ Most previous *in vitro* studies have relied on traditional microbiologic practices of culture and microscopy. Yet many bacterial species present in the vagina are not easily identified through cultivation but may play a role in disease, requiring molecular methods for detection. ^22^

Thus, molecular methods and human studies are required to capture data in real-life conditions and long-term exposures associated with menstrual cup use, and to characterize the cups’ microbial diversity and its interaction with the vaginal microbiome (VMB). These are especially needed in areas with poor water and sanitation facilities where users may be more susceptible to contamination due to poorer hygiene or inadequate cup washing and storage. By using 16S rRNA gene amplicon sequencing, this current study sought to comprehensively characterize the bacterial surface profile of the menstrual cup of cup users in a longitudinal sub-study nested within a large cluster randomized controlled trial among secondary schoolgirls in rural western Kenya. We examined cup use factors associated with the cup’s bacterial surface profile composition, including detection of putative pathogens, and correlation with users’ VMB, to understand the risk of contamination over two and a half years of follow-up.

## Methods

### Study design

Data for this study were generated from a prospective cohort of secondary school-aged adolescent girls enrolled in the Cups and Community Health (CaCHe) sub-study of the Cups or Cash for Girls (CCG) randomized controlled trial (described elsewhere ^23^^,^^24^). The CaCHe sub-study enrolled 436 girls attending one of six schools participating in the CCG trial that had been allocated to the menstrual cup intervention arm (n = 3 schools) or the usual practice control arm (n = 3 schools); comprising ~20% of girls in the menstrual cup and control arms of the CCG trial. Participants in the cup arm received one reusable menstrual cup (Mooncup©) with training on safe use and care directly following their baseline assessment at study start. Each menstrual cup was given with a dedicated breathable fabric carry bag for routine storage between uses and was distributed packed in the manufacturer’s box. Cups were replaced during the study if they were lost, stolen, or damaged. Participants in CaCHe were eligible if they were 14 years or older, had experienced 3 or more menses, were resident of the study area and attending a study school, were not visibly or reported pregnant, and had no disability precluding participation. All participants had to receive parent/guardian consent and provide written assent to participate.

### Data and specimen collection

CaCHe enrolled participants in May 2018 and conducted biannual study visits from 6-30 months of follow-up. At each visit, participants provided self-reported survey data, and self-collected vaginal swabs ^10^^,^^24^ for characterizing the VMB (via OMNIgene OMR-130, DNA Genotek swabs) and BV testing (determined via Nugent scoring ^25^). STI and HSV-2 were assessed annually at baseline and 12-months, but due to the COVID-19 pandemic the 24-month visit was missed and STI were collected at the 30-month visit. STI were measured via two additional swabs: *Chlamydia trachomatis* (CT) and *Neisseria gonorrhoeae* (NG) using GeneXpert (Cepheid, Sunnyvale, California, United States) and *Trichomonas vaginalis* (TV) (OSOM antigen detection, Sekisui, Lexington, MA, US), and blood samples were collected for HSV-2 testing (Kalon HSV-2 IgG ELISA; Kalon Biological Limited). ^23^

For girls in the menstrual cup arm, at the 6, 12, 18 and 30-month visit, a second OMNIgene swab was used to sample the inner and outer surface of their menstrual cup. Girls were asked to bring their menstrual cup to these visits; however, not all girls brought their cups due to various reasons (e.g. forgot at home, lost cup, currently menstruating and using the cup, etc). Cups were sampled by a study nurse or counsellor, by rubbing the swab over the entire inside and outside of the menstrual cup, including attention to the rim (details on swabbing procedures can be found in Extended data, cup swab protocol). Once collected, all swabs were placed in individual specimen collection bags and transported in cooler boxes with ice packs to the laboratory within ~4 hours for processing. All specimens collected for amplicon sequencing were stored at −80°C and shipped to University of Illinois Chicago Genomics Research Core for processing.

Self-administered surveys were completed in English or DhoLuo at every study visit on electronic tablets running the survey on Open Data Kit. Sociodemographic and behavioral data were collected, including participant age and indicators to assess girls’ menstrual patterns and hygiene behaviors, recent antibiotic use, and sexual activity. Household characteristics included household socioeconomic status, water source at home and household latrine type. Household socioeconomic status was measured following an absolute index score ^26^ that aggregated multiple household indicators and normalized them by asset type. This score was split into quintiles and dichotomised as ‘lower’ (quintiles 1-2) and ‘higher’ (quintiles 3-5), full details are described elsewhere. ^12^ Post-intervention, girls in the cup arm also provided information on their cup use (at their most recent period and ever), hand hygiene, and cup washing, dropping and storage. For participants whose menstrual cups were swabbed for microbiome sampling, the counsellor or nurse recorded cup condition, including indicators for use, damage, detritus, and smell on a cup observation survey. This nurse-administered form also asked girls to provide details on their patterns of cup use, cleaning, and storage.

Water, sanitation, and hygiene (WASH) facilities at study schools were assessed annually by a study officer to capture if school latrines had water and soap for handwashing, locks and privacy doors, and whether latrines were clean and in working condition. These variables comprised a WASH score (range 0-3) which assigned a point for directly observed (1) water for handwashing, (2) soap, and (3) an acceptable girl-to-latrine ratio of < 0-30 girls per latrine that was clean, had a slab, door, and roof in good condition, and had no strong odor or holes in the walls. WASH scores were dichotomised at the midpoint for analysis with ‘lower’ WASH scores indicating poorer WASH and ‘higher” scores indicating better WASH conditions at schools.

### Characterization of the cup and vaginal microbiomes

Methods of extraction, library preparation, and sequencing for the VMB have been published in detail. ^10^^,^^24^^,^^27^ We employed the same protocols for both the vaginal and cup swabs to enhance comparability and summarize briefly here, highlighting the differences. A two-stage PCR protocol using primers 357wF and 806nR (V3-V4 variable region) ^28^ was employed for PCR amplification and sequencing of microbial 16S rRNA genes. ^29^ The Illumina MiSeq instrument was used to sequence amplicons, with V3 chemistry (600 cycles). Microbiome bioinformatics were performed with QIIME2 2023.5. ^30^ Raw sequence data were checked for quality using FastQC and merged using PEAR. ^31^ Merged sequences were quality filtered using the q2-demux plugin. Sequences were also length filtered, wherein anything less than 400bp and above 500bp was removed from the dataset. Trimmed data was followed by denoising with DADA2 ^32^ (via q2-dada2). Primer adapter sequences were removed using *cutadapt* algorithm. ^33^ The contaminant removal software, *decontam,* ^34^ detected eight contaminants based on the prevalence of the ASVs in the reagent negative blank controls using default parameters. The contaminant ASVs were removed from the data. The ASV fasta sequences and DADA2 generated abundance tables were used for taxonomic classification using SpeciateIT with default parameters, ^35^ followed by generation of taxonomy count tables. Vaginal community state types (CST) were generated using nearest neighbor matching algorithm (VALENCIA ^36^). From the results, we identified that many ASVs that were classified within the family *Neisseriaceae* (but not *Neisseria gonorrhoeae*) from the SILVA reference database were being misclassified by SpeciateIT as *N. gonorrhoeae.* Thus, to avoid working with likely spurious classifications we removed these ASVs from downstream analysis. Observations with read counts < 1,000 (n = 7) were excluded from inferential analyses, and the mean/median number of reads per cup sample was 54,711/56,332. For vaginal samples, the minimum read counts for inclusion in this analysis was also 1,000, and the mean/median number of reads was 30,336/26,381.

### Statistical analysis

We conducted analyses to understand participant and use factors associated with cups’ bacterial surface profile composition (hereafter referred to as the ‘cup microbiome’ denoting the cup-associated microbial signature). The outcomes for targeted analyses were: (1) presence and relative abundance of putative pathogens ( *E. coli* and *S. aureus*); and (2) presence and relative abundance of coliform bacteria (aggregate of *Escherichia, Klebsiella, Citrobacter, Proteus* ^37^). We compared outcomes by participant characteristics using multivariable mixed effects regression models that accommodated the nested hierarchical structure of the data (observations nested in individuals nested in clusters) and binary (presence/absence) and continuous (relative abundance) outcomes. ^38^^,^^39^ Visit was included in all analyses for adjustment. Participant characteristics included those we believed could affect the vaginal microbiome (as a potential source of bacteria on the menstrual cup) or cup care and cleaning: age, duration of cup use, menstrual and hand hygiene, menstrual cup usage, washing, and storage behaviors, including the occurrence of dropping of the menstrual cup, current sexual activity, and vaginal CST. The distribution of BV and STI are reported descriptively. As previously shown in this cohort, ^24^ BV rarely occurred in the absence of vaginal CST-IV and therefore was not modeled as an explanatory factor. STI also was not modeled, as assumption of status as negative at interim visits would lead to inestimability. Household-level characteristics included socioeconomic status (SES), latrine type, and water source. We also examined school-level WASH scores.

To estimate how much of the menstrual cup microbiome (sink) may be contributed by the vaginal microbiome (source), we conducted fast expectation-maximization for microbial source tracking (FEAST). ^40^ To identify factors associated with the percentage of the cup microbiome that is contributed by the vaginal microbiome, we conducted mixed effects linear regression following methods outlined above. As a supplemental analysis, we calculated the Bray-Curtis similarity of the cup and vaginal microbiome. Where cup or vaginal taxa were not observed, they were assumed to be “0”. Sequence counts were natural log [ln(x + 1)] transformed prior to the generation of the Bray-Curtis resemblance matrix. To identify factors associated with Bray-Curtis similarity of the cup and vaginal microbiomes, we again applied mixed effects linear regression modeling. Data were analyzed in R (4.5.1) and Stata/SE (v17).

### Ethics statement

This study obtained approvals to conduct human subjects research from the Kenya Medical Research Institute’s Scientific Ethics Review Unit (KEMRI, SERU #3215), Liverpool School of Tropical Medicine’s Research Ethics Committee (LSTM, #15–005), and the University of Illinois at Chicago Institutional Review Board (UIC, #2017–1301). Written informed parental consent and written informed assent were obtained for all participants.

## Results

### Cup user characteristics

From 5 ^th^ October 2018 to 19 ^th^ January 2021, 172 participants (80.8%) from three secondary schools randomized to the menstrual cup intervention arm submitted 369 cup samples across four study visits, with 362 having at least 1,000 read counts ( Table 1). Of these, 28.5% had 1 (n=49), 37.2% had 2 (n=64); 25.6% had 3 (n=44); and 8.7% had 4 (n=15); representing 47.1% of study visits among menstrual cup arm participants. Median participant age at first cup sampling (6-month visit) was 17.1 years. Reported sexual activity increased from 10.7% at 6-months to 56.0% by 30-months. BV prevalence among girls submitting a cup sample increased from 9.3% at 6-months and 8.0% at 12-months, to 13.7% at 18-months and 26.0% at 30-months. STI prevalence increased from 13.3% at 12-months to 20% at 30-months. The proportion of participants with vaginal microbiota dominated by non-optimal CST-IV increased from 13.3% at 6-months, to 16.8% at 12-months, 19.1% at 18-months, and 38.0% at 30-months. Baseline characteristics, including age, socioeconomic status, BV/STI status, and vaginal CST did not significantly differ between participants who submitted cups and those who did not (Extended Data, Supplementary Table 1).

**Table 1.** Individual and household characteristics of participants who submitted ≥1 cup sample by study visit.

Characteristic ^1^	Month 6 (n = 74) n (%)	Month 12 (n = 110) n (%)	Month 18 (n = 128) n (%)	Month 30 (n = 50) n (%)
**Age, median (IQR)**	17.1 (16.3 – 18.2)	17.9 (17.1 – 18.8)	18.3 (17.3 – 19.2)	19.0 (18.3 – 20.1)
**Household wealth at baseline** [lower socioeconomic score vs higher]	51 (68.9)	77 (70.0)	98 (76.6)	39 (78.0)
**Vaginal CST**				
CST-I ( *L. crispatus* dominant)	27 (36.5)	52 (47.3)	53 (41.4)	14 (28.0)
CST-II ( *L. gasseri* dominant)	2 (2.7)	3 (2.7)	1 (0.8)	0 (0)
CST-III ( *L. iners* dominant)	32 (43.2)	33 (30.0)	44 (34.4)	16 (32.0)
CST-IV (mixed)	10 (13.5)	18 (16.4)	24 (18.8)	19 (38.0)
CST-V ( *L. jensenii* dominant)	1 (1.4)	2 (1.8)	5 (3.9)	0 (0)
Missing	2 (2.7)	2 (1.8)	1 (0.8)	1 (2.0)
**BV positive** (Nugent 7-10)	7 (9.5)	8 (7.3)	18 (14.1)	13 (26.0)
**STI positive** (composite of *C. trachomatis*, *N. gonorrhoeae*, and/or *T. vaginalis*) *	—	14 (12.7)	—	10 (20.0)
**HSV-2 seropositive**	10 (13.5)	20 (18.2)	21 (16.4)	8 (16.0)
**Sexually active**	8 (10.8)	53 (48.2)	59 (46.1)	28 (56.0)
**Taken antibiotics past 30 days**	10 (13.5)	31 (28.2)	23 (18.0)	7 (14.0)
**Used sanitary pads at last period**	64 (86.5)	87 (79.1)	117 (91.4)	41 (82.0)
**Used cloth at last period**	8 (10.8)	6 (5.5)	12 (9.4)	4 (8.0)
**Ever used a cup**	31 (41.9)	83 (75.5)	69 (53.9)	40 (80.0)
**Current cup use (at last period)**	27 (36.5)	68 (61.8)	58 (45.3)	35 (70.0)
**Cleaned cup at end of period**	9 (12.2)	68 (61.8)	25 (19.5)	37 (74.0)
**Any cup detritus**				
No deposits	58 (78.4)	64 (58.2)	99 (77.3)	33 (66.0)
Grainy or thick deposits	8 (10.8)	14 (12.7)	27 (21.1)	14 (28.0)
Missing	8 (10.8)	32 (29.1)	2 (1.6)	3 (6.0)
**Any malodor on cup**				
None	57 (77.0)	78 (70.9)	110 (85.9)	36 (72.0)
Blood or other	9 (12.2)	0 (0)	16 (12.5)	11 (22.0)
Missing	8 (10.8)	32 (29.1)	2 (1.6)	3 (6.0)
**Any damage to cup**				
None	66 (89.2)	75 (68.2)	119 (93.0)	41 (82.0)
Any damage ^§^	0 (0)	3 (2.7)	7 (5.5)	6 (12.0)
Missing	8 (10.8)	32 (29.1)	2 (1.6)	3 (6.0)
**Washed hands before inserting cup**				
Sometimes/Never	0 (0)	8 (7.3)	3 (2.3)	2 (4.0)
Always	11 (14.9)	41 (37.3)	27 (21.1)	38 (76.0)
Missing	63 (85.1)	61 (55.5)	98 (76.6)	10 (20.0)
**Rinsed cup between emptying/reinsertion**				
Always	7 (9.5)	65 (59.1)	21 (16.4)	31 (62.0)
Sometimes	0 (0)	5 (4.6)	7 (5.5)	2 (4.0)
Never	4 (5.4)	10 (9.1)	2 (1.6)	7 (14.0)
Missing	63 (85.1)	30 (27.3)	98 (76.6)	10 (20.0)
**School WASH score at baseline** [lower vs higher score]	37 (50.0)	66 (60.0)	76 (59.4)	31 (62.0)
**Water source at home**				
Surface water ^†^	39 (52.7)	52 (47.3)	58 (45.3)	23 (46.0)
Borehole	12 (16.2)	23 (20.9)	30 (23.4)	15 (30.0)
Rainwater	14 (18.9)	29 (26.4)	33 (25.8)	11 (22.0)
Pipe in house	1 (1.4)	4 (3.6)	6 (4.7)	1 (2.0)
Missing	8 (10.8)	2 (1.8)	1 (0.8)	0 (0)
**Latrine type at home**				
Flush toilet	7 (9.5)	11 (10.0)	14 (10.9)	1 (2.0)
Traditional pit ^α^	42 (56.8)	69 (62.7)	70 (54.7)	35 (70.0)
Ventilated improved pit	17 (23.0)	27 (24.6)	43 (33.6)	14 (28.0)
Missing ^‡^	8 (10.8)	3 (2.7)	1 (0.8)	0 (0)

Legend:

^1^ Variables are time-varying, unless specified as baseline measurement.

* STI was only tested annually and thus no results are presented at Month 6 or Month 18 visits.

^§^ Damage includes small wear and tear, splits in rim, cracks in bell, ragged/split tail, and general damage;

^†^ Surface water was any of the following: pond, lake, river, stream;

^α^ Includes n = 8 bush/field;

^‡^ Includes n = 1 “Other” (not specified). Abbreviations: CST = Community State Type; BV = Bacterial Vaginosis; STI = sexually transmitted infections; WASH = water, sanitation, and hygiene.

Ever using the cup increased from 42.7% to 80.0% over the study period, with reported current cup use at last period increasing from 37.3% at the 6-month visit to 70.0% by 30-months. Improvements in hygiene practices were noted over time: hand washing prior to cup insertion increased from 14.7% at 6-months to 76% at 30-months and cleaning the cup post period rose from 12% at 6-months to 74% at 30-months. Nonetheless, grainy or thick deposits on the cup were more visible as follow-up progressed with 10.7% of cups observed to have deposits at 6-months, increasing to 12.4% at 12-months, 20.6% at 18-months, and 28.0% at 30-months. Cup damage also increased by study visit, increasing from 2.7% at 12-months to 6.0% by 30-months.

### Characteristics of the cup microbiome

**Figure 1.**
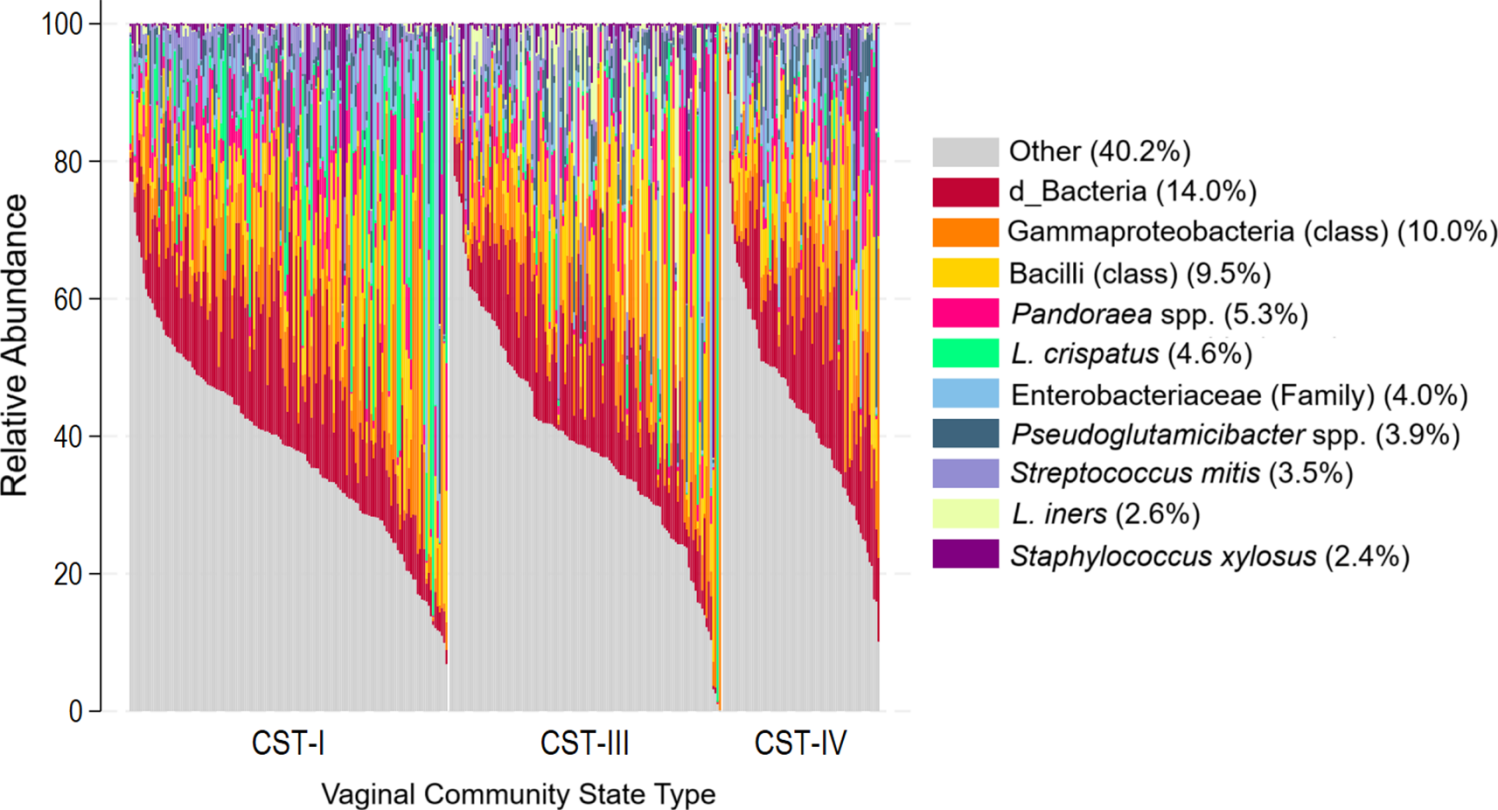
Relative abundance of top 10 cup microbiome taxa sorted by vaginal community state type. **Legend:** The y-axis shows the relative abundance of the taxa with the highest mean relative abundance with each bar representing one cup sample. Individual samples are sorted by the vaginal CST of the participant submitting the cup sample.

The top ten taxa with the highest relative abundance across all menstrual cup samples accounted for an average of 59.8% of total sequence reads ( Figure 1). Of these, a total of 7.2% of the relative abundance was related to taxa commonly associated with the vaginal microbiome: *L. crispatus* (4.6%) and *L. iners* (2.6%). Gammaproteobacteria (10.0%) and Bacilli (9.5%) were the taxa with the highest mean RA. Other prominent taxa included *Pandoraea* (5.3%), *Enterobacteriaceae* (4.0%), *Pseudoglutamicibacter* (3.9%) *, Streptococcus mitis* (3.5%) *,* and *Staphylococcus xylosus* (2.4%). An additional 14.0% of reads were classified as unknown bacterial taxa (d_Bacteria).

As shown descriptively in the stacked bar chart, there were marked taxonomic differences observed between cup samples taken from participants with optimal vaginal CST-I versus with non-optimal CSTs. For example, *L. crispatus* accounted for 7.22% of the mean relative abundance in the cup samples collected from participants with vaginal CST-I, while among participants with CST-IV this taxon only accounted for 0.62% (Extended Data, Supplementary Table 2). Among participants with vaginal CST-IV, *G. vaginalis* in the cup microbiome accounted for 4.27% of the mean relative abundance, compared to 0.26% mean relative abundance of cup microbiome for participants with vaginal CST-I. These differences are further quantified in subsequent modelling results.

### Factors associated with relative abundance and presence/absence of putative pathogens

For analyses using paired vaginal and cup microbiome data, there were 353 (97.5%) observations available. The mean relative abundance of *E. coli* and *S. aureus* was 0.09% and 0.36%, and they were present on 36% and 24% of cup observations, respectively. In multivariable analysis, the relative abundance of *E. coli* was higher on cups belonging to participants with vaginal CST-IV and those who were HSV-2 positive ( Table 2). Participants with vaginal CST-IV had 1.7 times the odds of *E. coli* presence on their cups relative to participants with other vaginal CSTs (OR = 1.70; 95% CI: 0.95-3.04, p = 0.075). The relative abundance of *E. coli* was also increased in cups with visible signs of damage. *E. coli* presence was associated with current cup use, with those reporting currently using their cups having 1.65 times the odds of *E. coli* being present on their cups relative to those not currently using their cups (OR = 1.65; 95% CI: 1.03-2.62, p = 0.036). Conversely, recent antibiotic use (last 30 days) was inversely associated with *E. coli* presence (OR = 0.47; 95% CI: 0.25-0.87, p = 0.016). Following a similar pattern as found with *E. coli,* the relative abundance of *S. aureus* was increased on cups that showed signs of damage.

**Table 2.** Factors associated with percent relative abundance and presence vs. absence of putative pathogens.

	*E. coli* Percent RA		*E. coli* Presence vs. Absence		*S. aureus* Percent RA		*S. aureus* Presence vs. Absence		Coliform bacteria Percent RA	
Characteristic	Beta	95% CI	OR	95% CI	Beta	95% CI	OR	95% CI	Beta	95% CI
**Age at baseline**	0.020	-0.011, 0.051	0.97	0.828, 1.13	-0.025	-0.133, 0.083	0.93	0.77, 1.12	-0.368	-0.737, 0.000 ^
**Vaginal CST-IV vs. Other**	0.115	0.004, 0.226 ^	1.70	0.95, 3.04+	0.343	-0.063, 0.749	1.04	0.53, 2.06	-0.600	-1.972, 0.772
**HSV seropositive**	0.210	0.082, 0.339 ^	1.22	0.67, 2.23	0.120	-0.322, 0.562	0.78	0.37, 1.63	-0.786	-2.32, 0.745
**Antibiotics (30 days)**	-0.052	-0.163, 0.060	0.47	0.25, 0.87 ^	-0.028	-0.437, 0.381	1.16	0.60, 2.23	0.923	-0.413, 2.26
**Current cup use**	0.073	-0.016, 0.161	1.65	1.03, 2.62 ^	0.068	-0.252, 0.388	0.65	0.38, 1.12	-0.329	-1.42, 0.761
**Damage to cup** ^§^	0.430	0.219, 0.642 ^	2.46	0.85, 7.07	1.535	0.749, 2.322 ^	2.24	0.68, 7.36	-0.544	-3.14, 2.05

Legend:

^^^ p-value ≤ 0.05; + 0.05 < p-value < 0.10;

^§^ Damage includes small wear and tear, splits in rim, cracks in bell, ragged/split tail, and general damage. Abbreviations: RA = Relative Abundance; OR = Odds Ratio; 95% CI = 95% Confidence Interval.

The mean relative abundance of coliform bacteria was 2.66% and presence was 94.2%. Consequently, we did not model the presence/absence of coliform bacteria. Notably, *Klebsiella* spp. was detected on 93.6% of cups. The relative abundance of coliform bacteria on cups showed an inverse relationship with participant age, with older girls’ cups having lower relative abundance of coliform bacteria on their cups. All other factors assessed were not associated with the presence or relative abundance of putative pathogens or other coliform bacteria (Extended Data, Supplementary Tables 3-5).

### Factors associated with similarity of cup and vaginal microbiome

**Figure 2.**
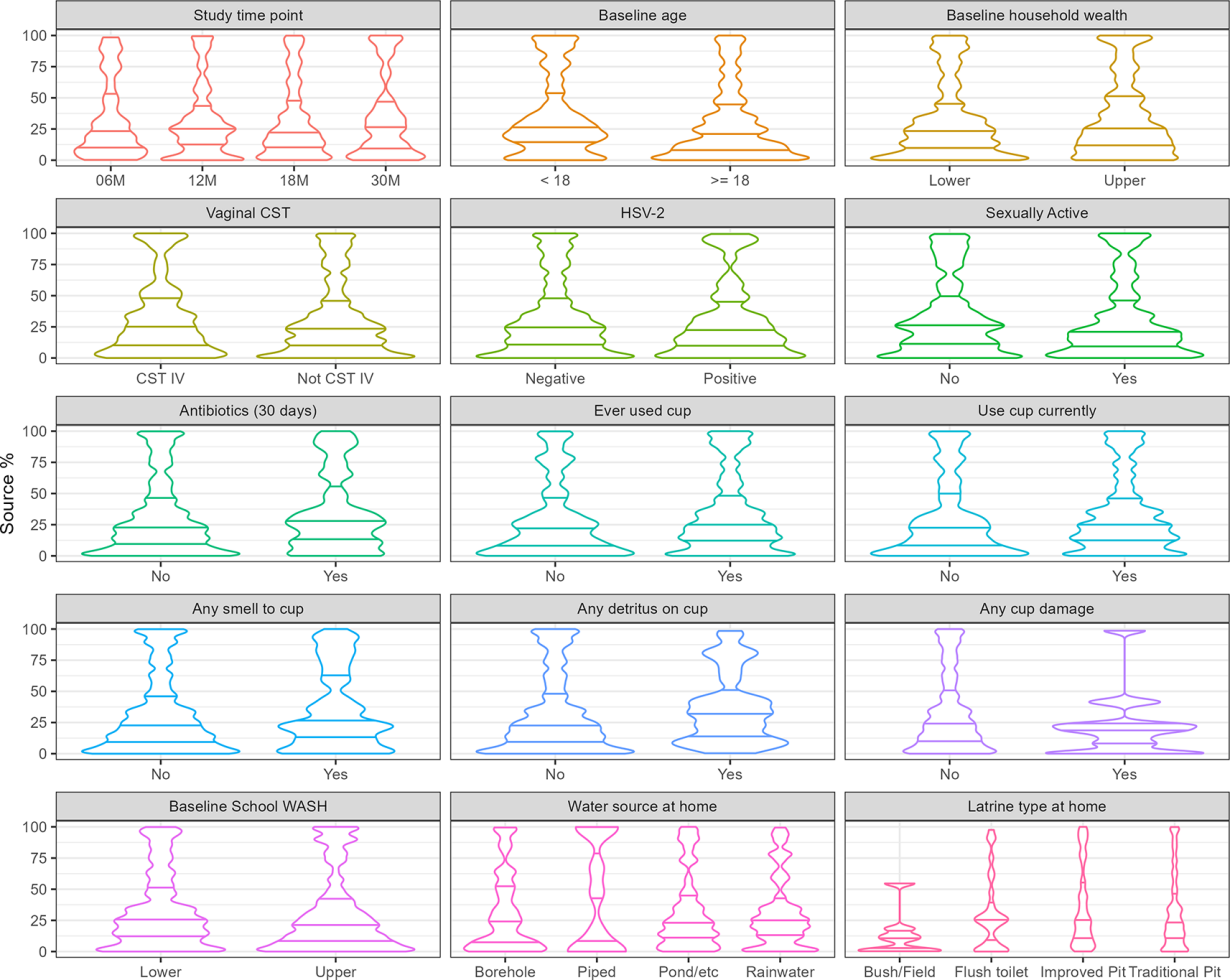
Percent of cup microbiome contributed by the vaginal microbiome: Distribution by covariates (n = 353). **Legend**: The distribution of the percent of the cup microbiome composition over covariates of interest is shown on the y-axis, with horizontal bars representing the median (middle bar), and lower and upper quartiles (lower and upper bars, respectively).

The median percent of the cup microbiome (“sink”) that was contributed by the vaginal microbiome (“source”) using all cup and vaginal microbiome taxa was 25.1% (IQR: 10.0%-45.9%). The distribution of the percent of the cup microbiome contributed by the vaginal microbiome over covariates is shown in Figure 2.

Mixed-effects linear regression identified participant age and damage to menstrual cup as significant predictors of the percent of the cup microbiome derived from the vaginal microbiome ( Table 3). Each additional year of age was associated with a 2.8% lower contribution of the vaginal microbiome to the cup microbiome (β = –0.028; *p* = 0.007), and cup damage was associated with a 13% lower vaginal taxa contribution (β = –0.130; *p* = 0.023) on the cup microbiome. Additionally, for participants who had vaginal CST-III there was a borderline significant but notable inverse association between the vaginal microbiome and cup microbiome, with more of the bacteria found on these cups coming from non-vaginal sources. Baseline vaginal CST was not associated with any source percentage and was not associated with subsequent contribution to the cup microbiome. There was no association between source-sink percentage and study visit, indicating that the patterns were stable. This analysis was repeated looking at only common taxa shared between the cup and vaginal microbiomes (n = 45) and when looking at all taxa excluding those with unknown bacteria (d_Bacteria). This analysis yielded the same factors of importance and direction of association (Extended Data, Supplementary Table 6). As a supplemental analysis examining factors associated with Bray-Curtis similarity of cup and vaginal microbiomes (Extended Data, Supplementary Table 7), only one factor was associated. Participants whose primary water source at home was piped water had significantly lower mean Bray-Curtis similarity (5.36%) compared to those using other water sources at home (9.23%-9.39%).

**Table 3.** Factors associated with percent of cup microbiome (sink) from vaginal microbiome (source).

	Percent of Cup (sink) microbiome sourced from vaginal microbiome β (p-value)
Visit	0.001 (0.836)
Age in years	**-0.028 (0.007)**
Lower Household wealth at baseline	0.029 (0.369)
Time-Varying Vaginal CST (vs. CST-I)	
CST-III	**-0.063 (0.076**)
CST-IV	-0.019 (0.658)
HSV-2 seropositive	-0.016 (0.700)
Sexually active	-0.019 (0.580)
Antibiotics used past 30 days	0.032 (0.439)
Pads used to manage menses	0.013 (0.785)
Cloth used to manage menses	-0.023 (0.715)
Ever used cup	0.035 (0.279)
Current cup use (at last period)	0.014 (0.677)
Any detritus on cup	0.031 (0.492)
Malodor on cup	0.052 (0.294)
Any damage to cup ^§^	**-0.130 (0.023)**
School WASH at Baseline	-0.028 (0.372)
School WASH at Follow-up	0.105 (0.113)
Water source at home (vs. surface water ^†^)	
Borehole	-0.009 (0.803)
Rainwater	-0.012 (0.744)
Pipe in house	0.095 (0.481)
Latrine type at home (vs. flush toilet)	
Bush	-0.138 (0.152)
Traditional pit	0.032 (0.600)
Ventilated improved pit	0.083 (0.192)

Legend: Bold denotes p-value ≤ 0.10;

^§^ Damage includes small wear and tear, splits in rim, cracks in bell, ragged/split tail, and general damage;

^†^ Surface water was any of the following: pond, lake, river, stream. Abbreviations: β = beta; CST = Community State Type; HSV-2 = Herpes Simplex Virus Type 2; WASH = Water, sanitation, and hygiene.

## Discussion

This study is the first to comprehensively characterize the menstrual cup microbiome using cups from a cohort of adolescent girls in western Kenya. We found that menstrual cups had a diverse microbiome, with a high proportion of soil- and water-associated taxa. Gammaproteobacteria and Bacilli together accounted for nearly 20% of relative abundance, and other bacteria widespread in soil and water such as *Pandoraea* ^41^ and *Pseudoglutamicibacter* ^42^ were also abundant, suggesting that much of the cup microbiome reflected exposure to environmental sources and handling. However, taxa specific to the vaginal microbiome (e.g., *L. crispatus, L. iners, G. vaginalis* ^43^^–^^45^) were also recovered from cups, indicating that despite reported cleaning practices, cups were not fully sterilized of host-associated bacteria.

While many of the most abundant taxa found on cups were not pathogenic, their presence reflects the potential impact of hygiene and environmental conditions on the cup microbiome. Some members of these taxa have been reported as opportunistic pathogens in clinical settings ^46^^,^^47^; however, their detection here may not indicate habituation, and may merely reflect environmental presence. Similarly, commensal or opportunistic organisms such as *Streptococcus mitis* and *Staphylococcus xylosus* were present on cups at low levels but are not generally associated with disease in healthy individuals. ^48^^,^^49^ Of note, no serious adverse events related to the menstrual cup, such as menstrual toxic shock syndrome, have been detected in the cohort, which was actively followed for 6.5 years (see Table 2 in Zulaika et al., 2023). ^12^

We found that the relative abundance and presence of *E. coli* were higher among participants with vaginal CST-IV and those testing HSV-2 positive. *E. coli* is frequently associated with Aerobic Vaginitis (AV), and known to play a role in vaginal dysbiosis, ^50^^,^^51^ with AV and BV co-infection relatively common. ^51^^,^^52^ Both *E. coli* and *S. aureus* were more commonly detected on damaged cups, suggesting that irregularities on the cup’s surface may allow for greater bacterial presence. The association between current cup use and increased odds of *E. coli* presence suggests that active use, rather than storage alone, facilitates transfer of putative pathogens. Recent antibiotic use was inversely associated with *E. coli*, indicating that the host’s microbiome also shapes the cup microbiome. These findings support that both host biological factors and cup condition are associated with putative pathogens found on menstrual cups. In the current analysis, water source at home and latrine type were not associated with the presence of *E. coli, S. aureus*, or coliform bacteria. Prior studies from this area have found that using stream water (vs. pond) and traditional or improved pit latrines (vs. flush toilets) increase the relative abundance of coliform bacteria; and that having piped water at home (vs. pond) and flush toilets (vs. traditional pit latrines) increased the odds of a healthy CST-I VMB among participants. ^37^

The high proportion of environmental taxa and presence of putative pathogens is notable for their potential ability to form biofilms, ^53^^,^^54^ and alter or exist at low pH, ^55^^,^^56^ potentially important for maintaining a healthy vaginal environment. However, one study looking at *S. aureus* biofilm formation on menstrual cups *in vitro* found that biofilm production was minimal and that existing guidelines for cup cleaning were adequate. ^19^^,^^57^ Moreover, the bioactivity of identified taxa on the cups remains unknown. It is possible that the cup microbiome composition is not only a function of the environmental and host-derived taxa, but also of further environmental selection on and due to the cup itself. However, without an assessment of the cup microbial community activity, it is difficult to further investigate this with marker gene data alone.

Using FEAST, we estimated that on average 25% of the cup microbiome was derived from the vaginal microbiome, with wide variability between individuals. These estimates tested the vaginal microbiome as the contributing source, though there may be multiple source environments, such as storage and handling conditions, which in our analysis are unmeasured and considered as an aggregate “unknown” convex. FEAST cannot determine directionality, and we are assuming directionality by estimating the VMB as source and cup microbiome as sink. Participant age and cup damage were significant predictors: older age and damaged cups were associated with a markedly lower proportion of vaginal taxa on the cup. These results may indicate that older participants were better at cleaning their cups, or that damaged cups accumulate more environmental bacteria, diluting the contribution from vaginal taxa. Alternatively, lower vaginal contribution to damaged cups may suggest less frequent use of these cups or less caretaking and cleaning in general. The Bray-Curtis similarity found low overlap between the cup and vaginal microbiomes, suggesting that the majority of bacteria detected on cups originate from environmental or handling-related exposures rather than directly from the vagina. Given these cups were swabbed during a school health day, the handling of cups in transit from home to school may have introduced additional environmental taxa, and inflated their representation compared to under normal use conditions.

### Limitations and future directions

This study had a few limitations to note. First, annotation relied on a vaginal-specific reference database (SpeciateIT) to facilitate comparability with the VMB samples. While this approach strengthened alignment between cup and vaginal data, it likely over-represented vaginal species and under-represented non-vaginal taxa, possibly inflating correlations between cup and vaginal samples. Additionally, some misclassification was evident; for example, Neisseriaceae sequences were mostly annotated as *Neisseria gonorrhoeae* (1.9% of total sequencing data). These sequences were removed from the downstream analysis to avoid over-speculation about a taxon of concern given *Neisseria* can be non-pathogenic in the environment. Expanded reference databases incorporating multiple other non-gonorrhea species, or more environmental and non-common vaginal taxa would improve specificity of the annotations, although we recognize this would be difficult to accommodate across all geographic locales and body sites. Second, our results reflect DNA detection rather than viable organisms; the presence of bacterial DNA on cups does not necessarily imply colonization, survival, or pathogenic potential. We also did not include a measure of days since last use, which could have influenced microbial detection. Future studies incorporating culture-based or metatranscriptomic approaches and indicators for time since last use, could better determine the viability, bioactivity, and persistence of cup-associated bacteria.

Third, many girls did not bring their cups to school and a number of girls who did reported never using their cup. However, samples derived from these cups were still included as these cups often showed signs of wear, indicating girls’ reporting of cup use may have been poor. Fourth, no self-reported cleaning practices were associated with putative pathogens or cup-vaginal similarity, which may reflect participant overreporting of cup cleaning practices or could support that cup contamination came from cup handling and storage conditions. It is possible that girls cleaned their cups more thoroughly just before use at the start of menses, and that the cup condition we captured was not reflective of the cup’s condition during use. Alternatively, it may be related to skip errors in the survey design which contributed to missing data on hygiene practices that we were unable to model. We also note that at the 6- and 18-month visits, the proportion of cup users appeared to be lower due to abbreviated question sets at these interim time points, while annual time points userd a longer question series to capture cup use behaviors. Future studies could consider direct observation of cleaning practices. To identify sources of environmental contaminants, one solution may be to characterize the microbiome of the environment in which the cups are cleaned and stored. Swabbing unused cups as negative controls could help to understand which bacteria were present on cups prior to first use straight out of the packaging versus after storage and acquired from the surrounding environment. Additionally, future approaches should also identify effective interventions to support cup cleaning, such as sustainable clean water approaches (e.g., solar water disinfection, ^58^^–^^60^ or chlorination of drinking water ^61^). Cup programs should identify and evaluate alternative storage containers (e.g. ventilated plastic or silicon cases, UV-sterilizing boxes, moisture-resistant pouches) to limit contamination and environmental exposures, alongside repeated user trainings on cup cleaning and safe care. ^19^ Water, sanitation, and hygiene infrastructure upgrades, including soap provision for handwashing and access to safe water and improved latrines, are also needed to ensure cup safety.

## Conclusions

This study characterized the menstrual cup bacterial surface profile of participants using cups for menstrual hygiene in a randomized controlled trial. Over 30 months of active surveillance, no adverse events were observed in relation to cup use, despite indication that cups were not fully sterilized. While environmental taxa accounted for the majority of the cup bacterial community composition, the detection of vaginal species such as *L. crispatus* demonstrates that cup cleaning practices did not fully eliminate host-derived bacteria. Presence of putative pathogens was associated with vaginal dysbiosis, infection status, and cup damage, while source–sink analyses demonstrated that both age and cup condition influenced the degree of vaginal contribution to the cup microbiome. These findings reinforce the need for interventions that support designing improved tools for cup cleaning and storage, access to clean water, and appropriate sanitation and hygiene facilities. We recommend that future cup programs integrate improved storage containers and conduct repeated trainings on cup cleaning and care to maximize the safety and acceptability of menstrual cups as a sustainable menstrual health option.

## Data Availability

The raw sequence data (FASTQ files) from this study are deposited in the National Center for Biotechnology Information (NCBI) Sequence Read Archive (SRA), under BioProject identifier PRJNA746243. Supplemental tables with additional results from this study and the menstrual cup microbiome swabbing protocol are available on figshare.com open access data repository ( https://doi.org/10.6084/m9.figshare.30334087.v1; and https://doi.org/10.6084/m9.figshare.30311773.v1, respectively). Participant level data is available upon request. This study was conducted with approval from the Kenya Medical Research Institute (KEMRI) Scientific and Ethics Review Unit (SERU), which requires that de-identified data from any Kenya-based study be released only after receipt of written KEMRI SERU approval for additional analyses. Application forms and guidelines can be accessed at https://www.kemri.org/seru-overview or by contacting seru@kemri.org.

## Extended data

### CaCHe: Cup microbiome study - Supplemental tables

Title: Supplemental tables for characterizing menstrual cup microbiomes and their association with user characteristics and vaginal microbiomes in an adolescent cohort from western Kenya

Name of the repository: figshare

DOI: https://doi.org/10.6084/m9.figshare.30334087.v1

License: CC BY 4.0

### Cup swabbing protocol

Title: Standard General Operating Procedure for Swabbing Menstrual Cups

Name of the repository: figshare

DOI: https://doi.org/10.6084/m9.figshare.30311773.v1

License: CC BY 4.0
